# Spatio-Temporal Pattern and Meteo-Climatic Determinants of Visceral Leishmaniasis in Italy

**DOI:** 10.3390/tropicalmed7110337

**Published:** 2022-10-29

**Authors:** Giovenale Moirano, Marta Ellena, Paola Mercogliano, Lorenzo Richiardi, Milena Maule

**Affiliations:** 1 Cancer Epidemiology Unit and CPO-Piemonte, Department of Medical Sciences, University of Torino, 10126 Torino, Italy; 2 Post Graduate School of Medical Statistics, Department of Public Health and Paediatrics, University of Torino, 10126 Torino, Italy; 3 Department Environmental Sciences, Informatics, and Statistics, Università Ca’Foscari Venezia, 30172 Mestre, Italy; 4 Regional Models and Geo-Hydrological Impact Division, Fondazione Centro Euro-Mediterraneo Sui Cambiamenti Climatici (CMCC), 81100 Caserta, Italy

**Keywords:** environmental epidemiology, meteo-climatic impacts, spatio-temporal epidemiology, visceral Leishmaniasis

## Abstract

Historically, visceral leishmaniasis (VL) in Italy was constrained to Mediterranean areas. However, in the last 20 years, sand fly vectors and human cases of VL have been detected in northern Italy, traditionally classified as a cold area unsuitable for sand fly survival. We aim to study the spatio-temporal pattern and climatic determinants of VL incidence in Italy. National Hospital Discharge Register records were used to identify incident cases of VL between 2009 and 2016. Incident rates were computed for each year (N = 8) and for each province (N = 110). Data on mean temperature and cumulative precipitation were obtained from the ERA5-Land re-analysis. Age- and sex-standardized incidence rates were modeled with Bayesian spatial and spatio-temporal conditional autoregressive Poisson models in relation to the meteo-climatic parameters. Statistical inference was based on Monte Carlo–Markov chains. We identified 1123 VL cases (incidence rate: 2.4 cases/1,000,000 person-years). The highest incidence rates were observed in southern Italy, even though some areas of northern Italy experienced high incidence rates. Overall, in the spatial analysis, VL incidence rates were positively associated with average air temperatures (β  for 1 °C increase in average mean average temperature: 0.14; 95% credible intervals (CrI): 0.01, 0.27) and inversely associated with average precipitation (β for 20 mm increase in average summer cumulative precipitation: −0.28, 95% CrI: −0.42, −0.13). In the spatio-temporal analysis, no association between VL cases and season-year specific temperature and precipitation anomalies was detected. Our findings indicate that VL is endemic in the whole Italian peninsula and that climatic factors, such as air temperature and precipitation, might play a relevant role in shaping the geographical distribution of VL cases. These results support that climate change might affect leishmaniasis distribution in the future.

## 1. Introduction

Visceral leishmaniasis (VL) is a vector-borne disease with a broad global occurrence, caused by parasites belonging to the Leishmania genus [1]. VL is usually diagnosed clinically among subjects that present irregular fever, anemia, leukopenia, and hepatosplenomegaly. Whereas most immunocompetent individuals develop few or no symptoms after Leishmania infection, pediatric, old, and immunocompromised subjects can develop the visceral form of the disease, which is typically fatal if untreated [2].

Leishmania parasites are transmitted to human hosts by the bite of infected phlebotomine sand flies. Worldwide, the two most important putative agents of VL, *Leishmania donovani* and *Leishmania infantum*, are estimated to cause up to 400,000 VL cases and up to 40,000 deaths per year [3,4]. While *L. donovani* is found in the tropical areas of Africa and Asia and its transmission is likely anthroponotic, with sand flies transmitting the parasite from human to human, *L. infantum* is found in Europe and in South America and its transmission is zoonotic, with sand flies transmitting the parasite usually from canine reservoirs to humans [5]. In Europe, endemic transmission of *L. infantum* is associated with the geographical distribution and abundance of competent vectors, mainly represented by *Phlebotomus perniciosus* [6,7,8].

The geographical distribution of phlebotomine sand flies is affected by several environmental parameters, such as elevation, terrain slope and roughness, land use, vegetation, air temperature, precipitation, and humidity [9,10]. For instance, air temperature can influence both the distribution and the seasonal activity of the sand flies, as well as the parasite incubation time within the vectors [11,12]. Consistently, cases of both canine and human leishmaniasis have been historically reported in the Mediterranean areas of Europe, characterized by hot summers and mild winters, with Albania, Georgia, Greece, Italy, and Spain contributing to more than 80% of European cases [6]. However, over the last decades, the pathogen and its vectors have been found to expand geographically. The reported expansion of the parasite has been suggested to be linked with the vector populations range expansion in response to altered meteo-climatic conditions caused by climate change [13,14]. For example, sand fly vectors have been recently recorded in some countries of central Europe, such as Germany and Belgium [15,16]. In addition, autochthonous cases of human VL have been observed since the 1990s in areas characterized by continental climate and historically considered non-endemic, such as northern Italy [17,18]. In this context, spatial and spatio-temporal epidemiology offer a well-validated methodology for studying whether the geographical and temporal distribution of a disease can be explained by environmental, social, and meteo-climatic determinants. With the current study, we aimed to study the spatio-temporal pattern and meteo-climatic determinants of VL incidence in Italy between 2009 and 2016, applying Bayesian spatial and spatio-temporal models.

## 2. Materials and Methods

### 2.1. Study Area

Italy has approximately 60 million inhabitants and a population density of 200 people per square kilometer. Population density is heterogeneous, with the Po Valley and the coastal areas being the most densely populated areas. Italy is administratively divided into 20 regions and 110 provinces. As the Italian peninsula extends from the Alps in the north to the center of the Mediterranean Sea in the south, it presents a variety of climate systems ranging from continental climate of the inland northern areas (Po Valley) to the Mediterranean climate profile of the coastal and insular areas [19]. The analysis of climate data recorded by the main national observation networks showed an increase of more than 1.1 °C in the average annual temperature in Italy over the period 1981–2010 (with 1971–2000 as a reference period). The last few years have been characterized by high temperature increases, with eight of the ten hottest years in the historical series from 2011 onward. In relation to precipitation, since the middle of the last century, the historic trend of cumulated precipitation shows a strong variability over the territory, with higher values along the Alps and pre-Alps areas, and the lowest average in southern Sicily, Puglia, southern Sardinia, Valle d’Aosta, and Alto Adige [20,21].

### 2.2. Data Collection and Management

#### 2.2.1. Health Data

VL-related hospitalizations were identified from the national Hospital Discharge Register (HDR) records of the Italian Ministry of Health–Directorate General of Health Programming. The hospital discharge record is a synthesis of the patient’s medical history, including demographic data (i.e., age, gender, province of residence, etc.) and clinical information (the principal diagnosis and up to five secondary diagnoses, coded with the International Classification of Diseases, 9th Revision, Clinical Modification, ICD 9 CM). In this database, each hospitalized patient is identified by an anonymous ID code. Hospital discharges for leishmaniasis (first three digits of ICD 9 CM = “085” in any diagnostic position) recorded between 1 January 2007 and 31 December 2017 (11 years) were selected. Since disease relapses are frequent and VL patients can be repeatedly admitted to the hospital, we used the ID code to identify only the first VL-related hospitalization of each subject. We included all the subjects with at least one hospital discharge for visceral leishmaniasis (ICD 9 CM = “085.0”) and unspecified leishmaniasis (ICD 9 CM = “085.9”), excluding cutaneous and mucocutaneous leishmaniasis (ICD 9 CM = “085.1”, “085.2”, “085.3”, “085.4”, “085.5”). In addition, we excluded subjects with a foreign nationality because VL is a rare disease in Italy and almost all identified VL cases with foreign nationality were citizens of countries endemic for VL and had acquired the infection outside Italy. Since clinical manifestations of VL can last several months and relapses are common, we excluded subjects that had been hospitalized for VL in 2007 and 2008. Finally, we introduced a 5-month lag to define the year of cases occurrence, namely considering all cases diagnosed before the 31st of May of each year as deriving from an infection acquired the previous year. This was done because sand fly activity at the Italian latitudes occurs during summer months, from June to September [11], and the incubation period of VL usually varies from 1 to 8 months [22]. Thus, cases reported at the beginning of the calendar year are likely to derive from infections likely acquired during the previous year. This led to the inclusion of all cases whose first VL-related hospital admission occurred between 1 June 2009 and 31 May 2017. Population data stratified by province, sex, and age were obtained from the Italian National Statistical Institute [23]. A flow diagram representing the case identification procedure is shown in the Supplementary Materials (Figures S1 and S2). 

#### 2.2.2. Meteo-Climatic Data

Meteo-climatic data were obtained from the latest ERA5-Land database climate re-analysis [24]. ERA5-Land is a climate reanalysis produced by the European Centre for Medium-Range Weather Forecasts (ECMWF), which provides a consistent view of the evolution of the land variables over several decades, allowing an accurate description of the climate of the past. Specifically, ERA5-Land provides atmospheric and land-surface variables with spatial resolution of about 9 km × 9 km. We retrieved the daily mean temperature (*T*) and the daily cumulative precipitation (*P*) for the whole Italian area over the entire study period (2009–2016). Gridded daily data were averaged over the years of the study period by season (winter, spring, summer, and autumn) to obtain the seasonal mean temperature ($T_{j}^{n}$) and cumulative precipitation ($P_{j}^{n}$), with *j* varying over the geographic cells and *n* over the four seasons, for the entire period 2009–2016. The season averages were also computed for each year, obtaining the season-year mean temperature ($T_{jt}^{n}$) and season-year cumulative precipitation ($P_{jt}^{n}$), with *t* varying over the years from 2009 and 2016. Thus, $T_{jt}^{n}$ represents the mean temperature and $P_{jt}^{n}$ the cumulative precipitation for each season *n*, cell grid *j,* and calendar year *t*. These sets of variables, $\left(T_{jt}^{n},\;P_{jt}^{n}\right)$ and *(*$T_{j}^{n},P_{j}^{n}),$ were then used to compute corresponding seasonal anomalies, $\Delta T_{jt}^{n}$ and $\Delta P_{jt}^{n}$, for each season n, cell grid *j*, and calendar year *t*. We defined seasonal temperature anomalies as $\Delta T_{jt}^{n}=T_{jt}^{n}-T_{j}^{n}$, i.e., the absolute differences between the season-year specific mean temperature and the corresponding 2009–2016 seasonal average values. Similarly, we defined seasonal rainfall anomalies as $\Delta P_{jt}^{n}=P_{jt}^{n}-P_{j}^{n}$, i.e., the absolute differences between the season-year specific cumulative precipitation and the corresponding 2009–2016 seasonal average values. Finally, all meteo-climatic data referred to each grid *j* were spatially aggregated at the province level *i*, with *i* varying from 1 to 110, the total number of Italian provinces. However, given that the inhabitants are not distributed homogeneously within a province area, we computed the population-weighted average of the gridded meteo-climatic parameters using a population density grid with a 9 × 9 km resolution and the 2010 population density raster from the WorldPop project [25]. 

#### 2.2.3. Potential Determinants of VL Data

In addition to the meteo-climatic data, we selected factors that could possibly influence the distribution of VL to be included in the model as spatial predictors. HIV-related hospital admission rates were included as potential determinants since HIV infection and immunodeficiency is a well-known risk factor for symptomatic VL [26]. HIV-related hospital admission rates averaged over the 2009–2016 period were obtained for each province from the Italian “Health for All” database, which brings together different health indicators including demographics, health determinants, and health outcomes [27]. We also included two environmental covariates that might play a relevant role in defining the suitable habitat for sand fly species, namely the surface roughness and the normalized difference vegetation index (NDVI) [7,11,17,28]. Surface roughness, which describes the slope irregularity of the terrain, was obtained for each province from the 1 km × 1 km elevation raster. Roughness was calculated by finding the largest inter-cell elevation difference of a central cell and its surrounding cells. Second, we retrieved the NDVI, which is a graphical indicator that describes the vegetation status of a selected area, averaged over the entire study period (2009–2016). NDVI values, which range between −1 and 1, was used as a proxy of greenness and non-artificial land cover. Both NDVI and roughness values were aggregated at the province level to study their relationships with VL as population-weighted averages, as done for meteorological parameters. 

### 2.3. Statistical Analysis

#### 2.3.1. Descriptive Analysis

We estimated the average VL incidence rates by age class (≤4, 5–14, 15–24, 25–44, 45–64, 65–74, and ≥75 years) and sex in the whole study area and for the 8 years of the study period. To evaluate the temporal trend of incident cases, we computed the age- and sex-standardized IR applying the direct standardization using the Italian census data of 2011 as reference population. To identify the areas at higher risk, an average standardized incidence ratio (SIR) for the whole study period and for each province was estimated as *SIR_i_ = Y_i_/E_i_*, where *Y_i_* is the observed number of VL cases in province *i*, and *E_i_* is the expected number of VL cases according to the age and sex distribution of the population living in the same province and the national VL rates. Province-specific SIRs were also estimated by each year of the study period as *SIR_it_ = Y_it_/E_it_*, where *Y_it_* is the observed number of VL cases in province *i* and year *t*, and *E_it_* is the expected number of VL cases according to the age and sex distribution of the population living in the province *i* in year *t*.

#### 2.3.2. Spatial and Spatio-Temporal Analysis

The spatial association between incident VL cases and each single climatic parameter was conducted to evaluate whether long-term climatic conditions could explain the spatial heterogeneity of VL incidence at the province level. These analyses were performed by fitting multiple Bayesian Poisson models, with spatially dependent random effects. Specifically, each model included the observed cases as outcome, one climatic parameter as the exposure of interest, NDVI, roughness, and HIV hospital admissions as covariates, the expected number of VL cases as an offset term, and a spatial random effect specified via the conditional autoregressive (CAR) method proposed by Leroux et al. [29,30] at the province level. The latter depends on two parameters: $\tau_{S}^{2}$, the variance of the spatial random effect, and ρS, which, spanning from 0 to 1, models the strength of spatial autocorrelation among neighbouring provinces. When ρS = 1, the model reduces to an intrinsic conditional autoregressive model (ICAR), while when ρS = 0, the model reduces to an independent mixed model. When ρS is not fixed, the model finds a balance between these two models by estimating the value of ρS. For each climatic variable, two sets of spatial models were fitted to the data, Model1, including an independent random effect at the province level (ρS fixed to 0), and Model2, including a spatial autocorrelated random effect at the province level (ρS left free). 

The spatio-temporal association between incident VL cases and each meteo-climatic anomaly ($\Delta T_{it}^{n}$*,* $\Delta P_{it}^{n}$) was conducted to evaluate whether, adjusting for long-term climatic conditions, season-year meteorological anomalies could explain the spatio-temporal heterogeneity of VL incidence at the province level in the 8 years under study. This analysis was performed by fitting multiple Bayesian spatio-temporal Poisson models separately for temperature and precipitation. This analysis represents the temporal extension of Model2. Each regression model included one province-year climatic anomaly as the exposure of interest, the corresponding average climatic parameter, NDVI, roughness, and HIV hospital admissions, as well as one spatial random effect, one temporal random effect, and a spatio-temporal interaction term. The spatial and temporal random effects were specified, respectively, with a province and year conditional autoregressive (CAR) effect proposed by Leroux, while the spatio-temporal interaction term was specified with a set of independent province-year random effects. More details about the spatial and spatio-temporal Bayesian models and prior specifications are reported in the Supplementary Materials. 

For all the Bayesian models, the parameters were estimated by generating two independent Markov chains of 250,000 samples each, using the CARBayes and CARBayesST R packages [31,32]. After burning the first 50,000 initial samples, chains were thinned by a factor of 10, leading a total of 20,000 samples per chain. The 40,000 posterior samples obtained were then summarized as median values and 95% credible intervals (2.5 and 97.5 percentiles). The relationship between the outcome and the meteo-climatic variables were summarized as Poisson regression beta coefficients, indicating the average change in log-expected counts as the response to a unit increase in the climatic parameter (1 °C for temperatures, 20 mm for precipitation).

## 3. Results

### 3.1. Descriptive Analysis

**Figure 1:**
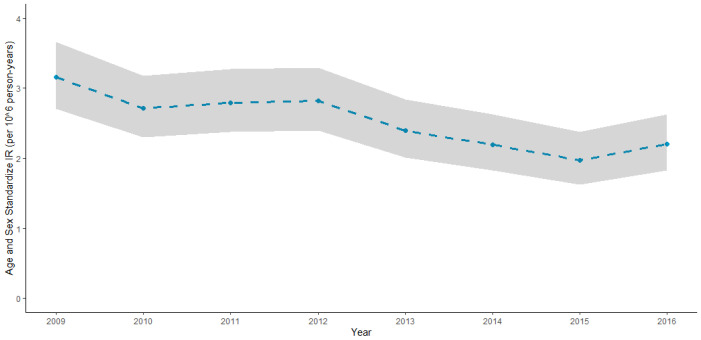
Year-specific VL standardized incidence rates between 2009 and 2016. Age and sex standardized IR are expressed per 10^6^ person-years and were computed with direct standardization using the Italian census data of 2011 as reference population. Estimates are represented as dashed lines, while the 95% CI bands are represented as shaded areas.

**Figure 2:**
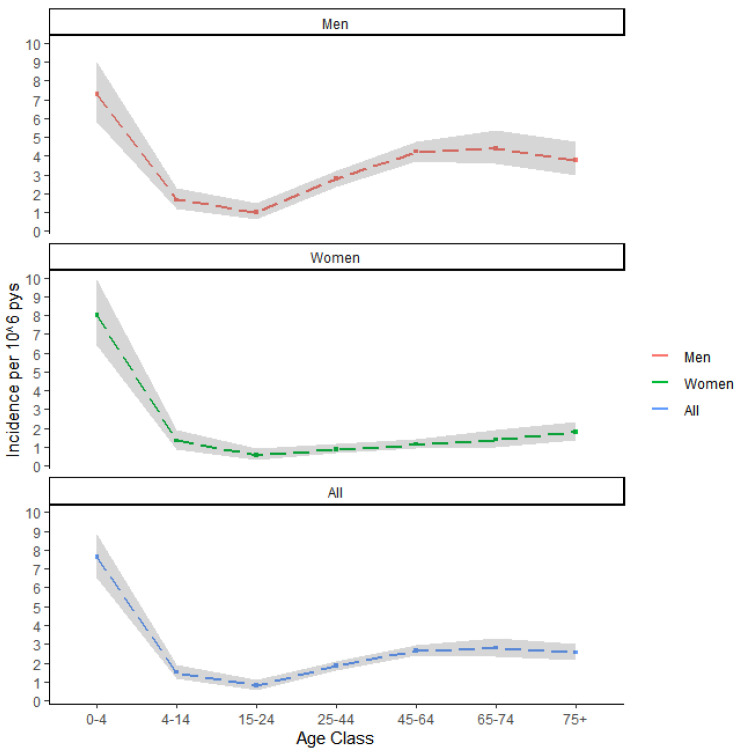
Average age- and sex-specific VL incidence rates over the 2009–2016 period. Incidence rates are expressed per 10^6^ person-years and were computed using the Italian census data of 2011. Estimates are represented as dashed lines, while the 95% CI bands are represented as shaded areas.

In the 8 years of study (2009–2016), 1126 VL cases were identified among Italian residents, corresponding to a crude incidence rate (IR) of 2.43 cases per 10^6^ person-years (95% CI 2.23–2.51). We observed a slight decrease in incidence rates over time, with a more pronounced decrease in the first half of the study period, and a stabilizing trend in the second half (see Figure 1). VL incidence rates show two peaks in the age groups of 0–4 years (IR: 7.63 cases per 106 person-years, 95% CI: 6.52–8.87) and 65–74 years (IR: 2.79 cases per person-years, 95% CI: 2.35–3.30), with the second peak sensibly more pronounced among men than women (see Figure 2).

### 3.2. Spatial and Spatio-Temporal Analysis

**Figure 3:**
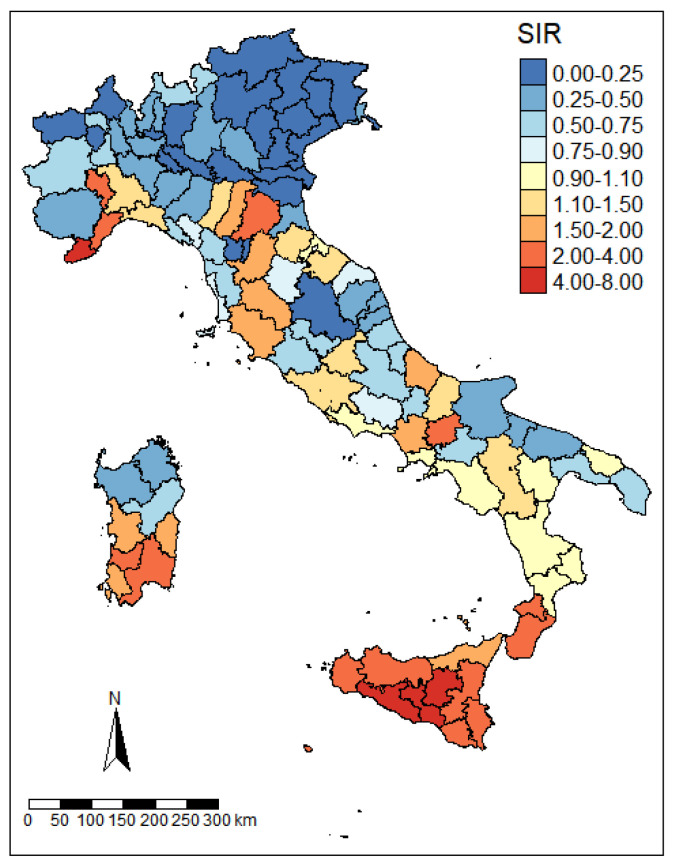
VL standardized incidence rates by Italian provinces (2009–2016)**.** Choropleth map representing VL standardized incidence ratio (SIR) at province level for the 2009–2016 period. Provinces with higher risk than the Italian average (SIR > 1.10) are represented in shades of red, while provinces with lower risk (SIR < 0.90) are represented in shades of blue.

The overall geographical distribution of VL risk during the entire study period is shown in Figure 3. The highest excess of risk was observed in the major islands and in southern Italy, although high SIRs were also observed in some coastal and inland areas of northern Italy. Standardized Incidence Ratios for VL for each year and province under study are reported in the Supplementary Materials (Figure S3).

In the spatial analysis, when average VL incident cases were studied in relation to climatic parameters, we observed a positive association between 2009–2016 average temperatures and VL incidence, both in Model A, assuming independence of random effects, and in Model B, modeling the spatial correlation between neighboring provinces (see Table 1). The strongest effect was detected for 1 °C in average summer temperatures (Model A | β: 0.24, 95% credible interval (CrI): 0.14, 0.37; Model B | β: 0.14 , 95% CrI: 0.01, 0.27). Concerning precipitation, we observed an inverse association between the 2009–2016 average cumulative precipitation and VL incidence. The strongest effect was found for a 20 mm increase in average spring cumulative precipitation (Model A | β: −0.37, 95% CrI: −0.47, −0.23; Model B | β: −0.28, 95% CrI: −0.42, −0.13). Spatio-temporal maps for yearly SIRs are shown in the Supplementary Materials (Figure S3). On the contrary, in the spatio-temporal analysis, no association between VL cases and season-year specific temperature and precipitation anomalies were detected (see Table 2).

## 4. Discussion

In this study, we described the spatio-temporal pattern of VL incidence in Italy during the period 2009–2016 using data from the national HDR records. The high incidence rates observed in subjects under 4 years of age (7.63 cases per 10^6^ person-years) are consistent with results from previous studies conducted in southern Europe (Spain, France, and Portugal) and confirm the typical epidemiological pattern of VL in the Mediterranean basin, where the *L. infantum* agent predominantly causes clinical disease in the pediatric population [33,34,35,36].

VL incidence rates show a high degree of heterogeneity across Italy, exhibiting an increasing gradient from north to south, and with the highest values in the Sicilian Island (Agrigento Province average IR: 16 cases per 10^6^ p-y). The geographical distribution of cases also shows an increasing gradient from east to west at all latitudes, with the highest incidence among the provinces of the Tyrrhenian coast. Interestingly, this east–west gradient has also been seen in the spatial distribution of canine leishmaniasis and might reflect different climatic and orographic characteristics [37]. For what concerns low-risk areas, the provinces reporting no VL cases during the study period (N = 10) are all located in northern Italy. However, some hotspot areas were observed in both coastal and inland northwestern provinces (Liguria and South Piedmont), and among northeastern Apennine provinces (Emilia-Romagna). These findings are consistent with previous studies that reported the presence of sand fly vector and cases of canine leishmaniasis, as well as cases of human VL in the same areas [17,37,38,39].

The occurring changes in climatic conditions, together with the relocation of infected dogs from southern kennels to the north of Italy, have been suggested to be among the potential causes of the geographical shift observed in the human and canine diseases [40]. With this study, we aimed to test the role of climatic conditions in shaping the VL epidemiology, by modeling the spatial and temporal distribution of human cases. Given the sparsity of the disease in Italy, we first assessed the spatial association between the cumulative incident of VL cases in relation to climatic parameters, averaged over the entire study period. We observed a positive association between VL incidence and seasonal air temperatures, with the strongest effect for summer temperatures, and an inverse association with seasonal precipitation, with the strongest effect during spring. The first result confirms the hypothesis that the air temperatures play a role in shaping the geographical distribution of sand fly vectors. Increased temperatures have been found to shorten the vector development time and to increase the vector biting rate, the vectorial capacity, and the parasite replication within the vector [41,42]. Previous studies have also shown a positive spatial association between air temperatures and VL in different areas of the world, such as South America, Asia, eastern Africa, and southern Europe [43,44,45,46]. On the contrary, studies assessing the effect of increased rainfall on VL incidence cases reported conflicting results, with both positive [45,47,48,49] and negative associations [50,51] around the world. Precipitation and soil moisture might play an important role in the sand flies’ life cycle. However, compared to mosquito vectors, sand flies do not have an aquatic life stage [52], and even if optimal soil moisture is required for egg survival, heavy rainfall can limit flight activity, reduce resting site availability, and cause the death of immature stages [53]. It is thus likely that the effect of rainfall depends on the local characteristics of the geographical area under study, modifying the complex interplay between temperature, humidity, precipitation, and sand fly vectors population. Overall, our study suggests that long-term climatic parameters are important in delimiting the boundaries of the area where vectors and parasite can thrive and, thus, identifying the areas at high risk of VL transmission. These results agree with previous studies that have suggested that the complexity of VL transmission is impacted by environmental factors. Like other vector-born infections, leishmaniasis is potentially impacted by global climate change and that distribution of sand fly vectors is expanding because of global climate change in specific regions such as Europe, Americas, Eastern Africa and Southeast Asia [1,2]. 

In the spatio-temporal analysis, no clear associations between VL cases and year-specific temperature or precipitation anomalies were observed. These findings do not support the idea of a short-term effect of meteorological anomalies on VL incidence. However, the lack of association could also be due to the limited statistical power of the current study, caused by the small number of incident cases and the relatively short period of observation (8 years). Unfortunately, we could not extend our period of observation because HDR records were not available before 2009. 

There are some important aspects and limitations that need to be considered when interpreting our findings. Only VL cases requiring hospitalization were included. Moreover, systematic differences in the likelihood of VL diagnosis might occur in different regions of Italy. In addition, considering that hospitalization occurs after a variable time interval from the onset of symptoms, the use of HDR records for case identification has hindered the definition of the exact time of infection. This misclassification might have diluted potential associations in the spatio-temporal analysis between meteorological anomalies and VL incidence. Taking into account the limitations described, our study has provided evidence of an association between climatic parameters and VL incidence, integrating several data sources including environmental, meteo-climatic, and epidemiological data at the national level, and adopting a Bayesian approach that allowed adjustment for residual spatio-temporal correlation and spatially structured unmeasured confounders. To our knowledge, this is the first attempt to evaluate the role of meteo-climatic parameters in shaping the risk of human VL covering a broad area of southern Europe, such as the Italian peninsula, where *L. infantum* is actively circulating. In Europe, previous studies mainly targeted the effects of meteo-climatic parameters on the vector population dynamics or on the reservoir’s seroprevalence [7,8,11,40,54,55]. Although the study was conducted by considering a relatively short period of time, the spatial association between VL incidence and meteo-climatic parameters underscores the relevance of climatic determinants in the complex human–vector–parasite nexus and suggests that monitoring climate-sensitive diseases is a valuable tool for early detection of possible adverse health effects of climate change.

## 5. Conclusions

Our findings suggest that the long-term climatic parameters can play a relevant role in shaping the epidemiology of VL in southern Europe, with higher temperatures increasing VL incidence and higher precipitation decreasing it. On the other hand, we found no evidence of short-term effects of temperature and precipitation anomalies. However, the slow but steady increase in temperatures caused by climate change could influence the spread of VL by extending areas characterized by climatic conditions to be suitable for vector species’ survival and proliferation. 

## Figures and Tables

**Table 1:** Regression coefficients from the spatial models describing the association between the 2009–2016 VL incidence cases and climatic parameters values averaged over the 2009–2016 period.

	Model 1		Model 2		
	β (95% CrI)	$\tau_{S}^{2}\;$ (95% CrI)	β (95% CrI)	$\tau_{S}^{2}\;$ (95% CrI)	ρS (95% CrI)
**Mean Temperature (1 °C increase)**					
Winter	0.17 (0.09, 0.25)	0.52 (0.36, 0.77)	0.09 (−0.01, 0.19)	1.01 (0.66, 1.55)	0.75 (0.33, 0.95)
Spring	0.23 (0.12, 0.36)	0.53 (0.36, 0.79)	0.10 (−0.02, 0.24)	1.02 (0.67, 1.56)	0.81 (0.43, 0.96)
Summer	0.24 (0.14, 0.37)	0.50 (0.34, 0.75)	0.14 (0.01, 0.27)	0.97 (0.63, 1.50)	0.77 (0.37, 0.96)
Autumn	0.22 (0.13, 0.33)	0.50 (0.34, 0.74)	0.12 (0.00, 0.25)	0.97 (0.62, 1.51)	0.75 (0.27, 0.95)
**Cumulative precipitation (20 mm increase)**					
Winter	−0.06 (−0.18, 0.05)	0.64 (0.44, 0.95)	−0.07 (−0.18, 0.05)	1.06 (0.69, 1.63)	0.85 (0.60, 0.97)
Spring	−0.34 (−0.47, −0.23)	0.44 (0.30, 0.66)	−0.28 (−0.42, −0.13)	0.88 (0.55, 1.36)	0.66 (0.25, 0.92)
Summer	−0.23 (−0.30, −0.16)	0.39 (0.26, 0.59)	−0.21 (−0.31, −0.12)	0.74 (0.45, 1.20)	0.53 (0.15, 0.87)
Autumn	−0.19 (−0.28, −0.11)	0.50 (0.34, 0.75)	−0.14 (−0.24, −0.04)	0.94 (0.62, 1.46)	0.76 (0.41, 0.95)

Model1: estimates adjusted for NDVI, roughness and HIV hospital admissions obtained from the Leroux model with ρS = 0 (independent random effect); Model2: estimates adjusted for NDVI, roughness and HIV hospital admissions obtained from the full Leroux model with ρS left free (spatially correlated random effects). ρS: median values for beta coefficients referred to a unit increase in the climatic parameter (1 °C for temperatures and 20 mm for precipitation) from 40,000 posterior draw samples; 95% CrI: 95% credible intervals (2.5–97.5%) computed from 40,000 posterior draw samples.

**Table 2:** Regression coefficients from the spatio-temporal models describing the association between yearly VL incidence, yearly meteo-climatic anomalies over the 2009–2016 period.

	Season-Year Anomaly	Spatial Random Effect		Temporal Random Effect		Spatio-Temporal Interaction
	β (95% CrI)	$\tau_{S}^{2}$ (95% CrI)	ρS (95% CrI)	$\tau_{T}^{2}$ (95% CrI)	ρT (95% CrI)	$\tau_{I}^{2}$ (95% CrI)
**Mean Temperature (1 °C increase)**						
Winter	−0–04 (−0.13, 0.08)	0.96 (0.61, 1.50)	0.77 (0.34, 0.96)	0.01 (0.00, 0.04)	0.51 (0.03, 0.95)	0.10 (0.02,0.18)
Spring	0.01 (−0.12, 0.14)	0.98 (0.62, 1.52)	0.82 (0.46, 0.96)	0.01 (0.00, 0.04)	0.58 (0.04, 0.96)	0.09 (0.01,0.18)
Summer	0.01 (−0.10, 0.12)	0.92 (0.59, 1.44)	0.78 (0.36, 0.96)	0.01 (0.00, 0.04)	0.56 (0.03, 0.95)	0.09 (0.01,0.18)
Autumn	−0.06 (−0.21, 0.09)	0.93 (0.59, 1.46)	0.75 (0.29, 0.95)	0.01 (0.00, 0.04)	0.51 (0.03, 0.95)	0.09 (0.02,0.18)
**Cumulative Precipitation (20 mm Increase)**						
Winter	0.02 (−0.01, 0.06)	1.02 (0.65, 1.58)	0.86 (0.61, 0.97)	0.01 (0.00, 0.04)	0.51 (0.03, 0.95)	0.09 (0.01, 0.18)
Spring	−0.01 (−0.06, 0.4)	0.83 (0.52,1.33)	0.69 (0.27, 0.93)	0.01 (0.00, 0.04)	0.57 (0.04, 0.96)	0.10 (0.03, 0.18)
Summer	0.03 (−0.03, 0.08)	0.71 (0.43,1.16)	0.56 (0.16,0.89)	0.01 (0.00, 0.05)	0.57 (0.04, 0.96)	0.09 (0.02,0.18)
Autumn	0.01 (−0.02, 0.04)	0.95 (0.61, 0.97)	0.78 (0.43,0.95)	0.01 (0.00, 0.04)	0.56 (0.04, 0.96)	0.09 (0.01, 0.18)

β: median values for beta coefficients referred to a unit increase in the climatic parameter (1 °C for temperatures and 20 mm for precipitation) from 40,000 posterior draw samples; 95% CrI: 95% credible intervals (2.5–97.5%) computed from 40,000 posterior draw samples and adjusted for NDVI, roughness and HIV hospital admissions.

## Data Availability

Restrictions apply to the availability of these data. Data was obtained from the Italian Ministry of Health—Directorate General of Health Programming.

## Supplementary Materials

The following supporting information can be downloaded at https://www.mdpi.com/article/10.3390/tropicalmed7110337/s1: Figure S1: Flow chart for cases identification; Figure S2: Case identification and temporal relationship with meteorological anomalies; Figure S3: Standardized Incidence Ratios for VL for each year and province under study.
