# Supplementary material for: Spatio-Temporal Pattern and Meteo-Climatic Determinants of Visceral Leishmaniasis in Italy
Source: Trop Med Infect Dis. 2022 Oct 29;7(11):337. doi: 10.3390/tropicalmed7110337 (PMC9694427; doi:10.3390/tropicalmed7110337)
Supplement: Supplementary file 1 [file tropicalmed-07-00337-s001.zip › tropicalmed-1923900-supplementary.pdf]

Supplementary Materials

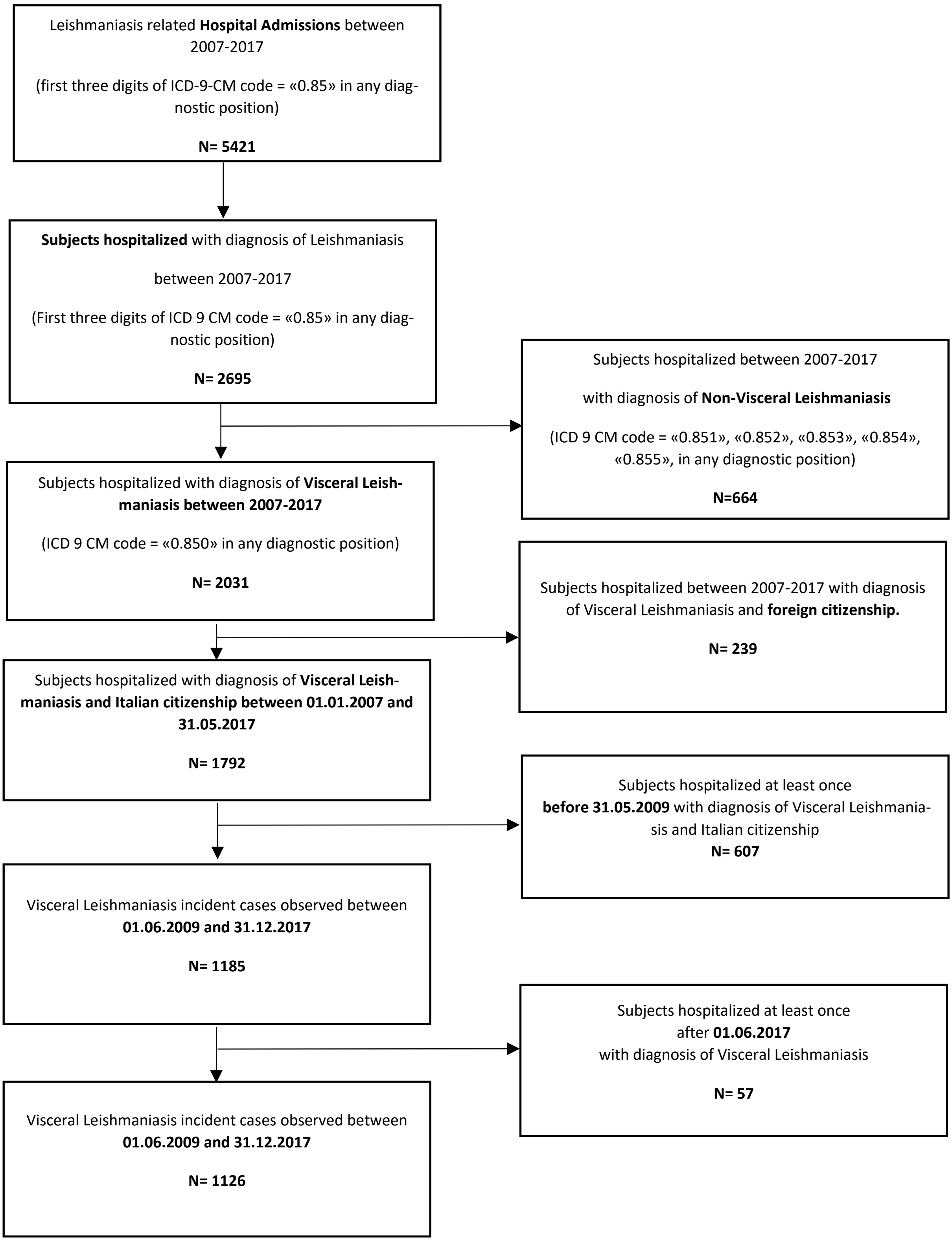

Figure S1. Flow chart for cases identification.

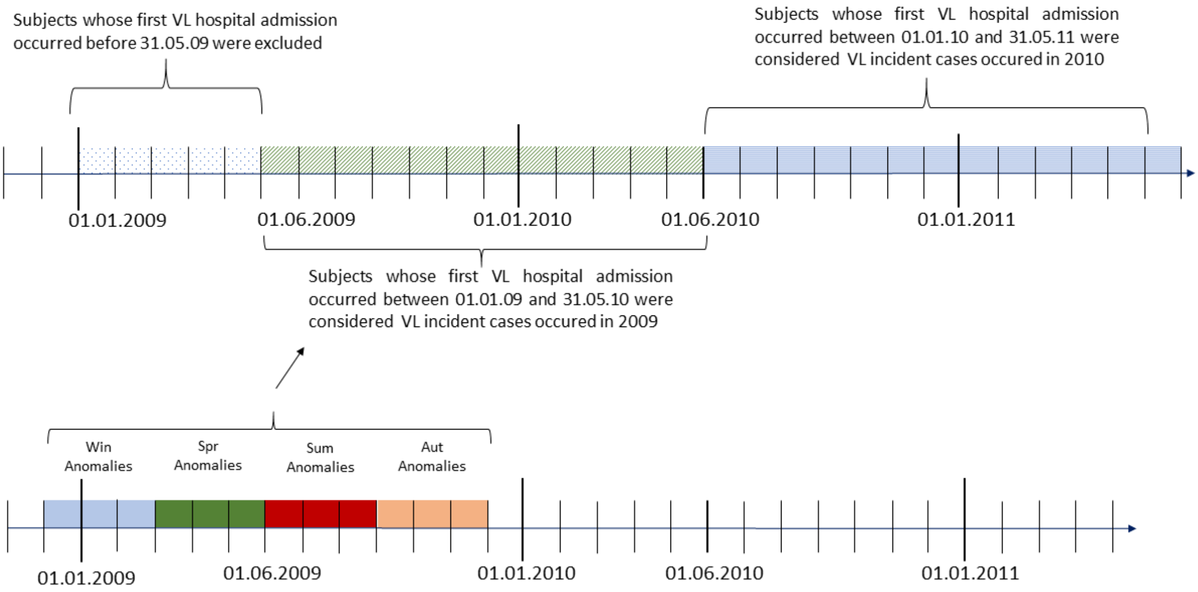

Figure S2. Case identification and temporal relationship with meteorological anomalies (example on years 2009-2011) for the spatio-temporal analysis.

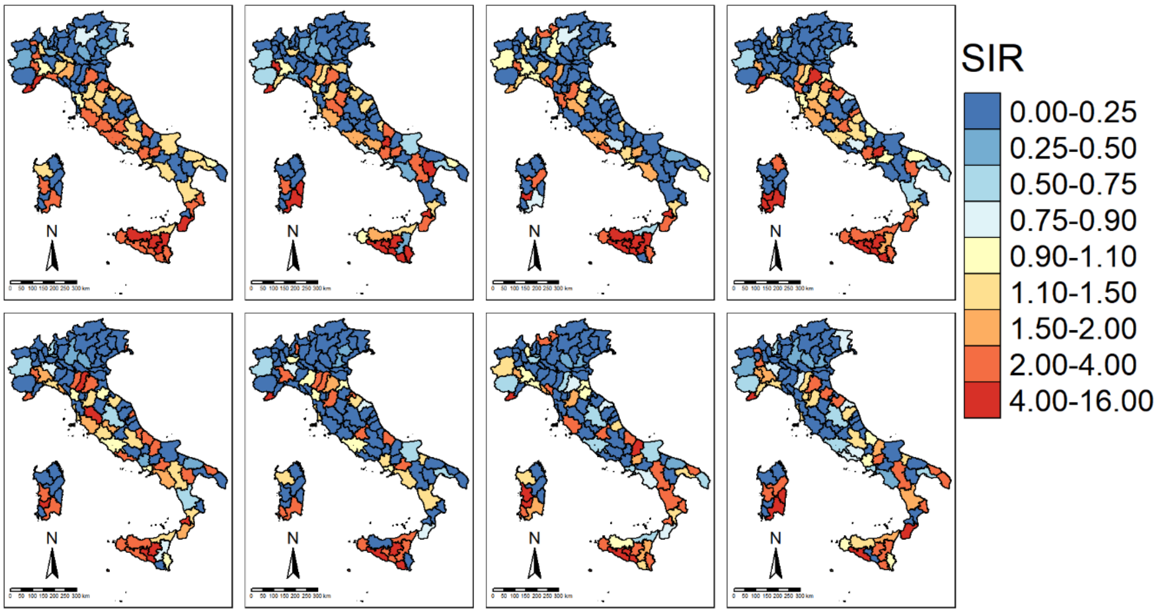

Figure S3. Standardized Incidence Ratios for VL for each year and province under study.

Description of the spatial model

The spatial association between incident VL cases and each single climatic parameter (T_i^n, P_i^n) was studied by fitting multiple Bayesian Poisson models, with spatially dependent random effects. Specifically, each model included the observed cases as outcome, one climatic parameter as the exposure of interest, NDVI, roughness, and HIV hospital admissions as covariates, the expected number of VL cases as an offset term, and a spatial random effect specified via the conditional autoregressive (CAR) method proposed by Leroux at province level [1,2]. The latter depends on two parameters: τ_S^2, the variance of the spatial random effect, and ρS, that models the strength of spatial autocorrelation among neighbouring provinces. When $\rho S = 1$, the model reduces to an intrinsic conditional autoregressive model (ICAR), while when $\rho S = 0$, the model reduces to an independent mixed model. When ρS is not fixed, the model finds a balance between these two models by estimating the value of ρS. For each climatic variable, two sets of spatial models were fitted to data, Model1, with $\rho S = 0$ (Independent Mixed Model), and Model2, where ρS was left free (Leroux Model).

Taking the 2009-2017 average of winter temperature as exposure of interest, the spatial distribution of VL cases for each province *i* was modelled as follows:

$$Y_i \sim Poisson\ (\mu_i)$$

$$ln(\mu_i) \sim ({T_i}^{Win})^T\ \beta_1 + Z_i^T\gamma + \varphi_i + ln(E_i)$$

Where μ_i is the expectation of the observed VL cases, ${T_i}^{Win}$ is the 2009-2016 average of winter temperatures, Z_i are baseline explanatory spatial covariates (HIV hospital admission rates, roughness, NDVI), E_i are the expected number of VL cases and φ_i is the set of spatial autocorrelated random effects. The spatial random effect, $\varphi = (\varphi_1, \ldots, \varphi_{110})$ depends on ρS parameter, ranging from 0 to 1, that models the strength of spatial autocorrelation among neighbouring provinces, defined by a binary neighbourhood matrix W taking value of 1 for each pair of provinces sharing borders.

The prior distribution for the spatial effect were specified as follow:

$$\varphi_i|\varphi_j\ , W \sim N\left(\frac{\rho S \sum_{j=1}^{I} w_{ij}\varphi_j}{\rho S \sum_{j=1}^{I} w_{ij} + 1 - \rho S}\quad , \quad \frac{\tau_S^2}{\rho S \sum_{j=1}^{I} w_{ij} + 1 - \rho S}\right)$$

With:

$$\tau_S^2 \sim InvGamma(1{,}0.01)$$

$$\rho S \sim Uniform\ (0{,}1)$$

Description of the spatio-temporal model

Each spatio-temporal regression model included one province-year climatic anomaly as the exposure of interest, the corresponding average climatic parameter, NDVI, roughness and HIV Hospital admissions, as

well as one spatial random effect, one temporal random effect and a spatio-temporal interaction term. Spatial autocorrelated random effects were modelled via the conditional autoregressive (CAR) method proposed by Leroux (ρS left free). This component captures the overall spatial random effect common to all time periods after adjusting for covariate effects. Temporal autocorrelated random effects were also specified via the conditional autoregressive (CAR) method proposed by Leroux. This temporal autocorrelated random effect captures the overall temporal trend common to all communities and depends on two parameters: τ_t^2, the variance of the temporal random effect, and ρT, that models the strength of temporal autocorrelation among consecutive years. Lastly, the spatio-temporal interaction component is a set of independent space–time effects interactions [3].

Taking winter temperature anomaly as exposure of interest, the spatio-temporal distribution of VL cases for each province *i* and period *t* was modelled as follows:

$$Y_{it} \sim Poisson\ (\mu_{it})$$

$$ln(\mu_{it}) \sim\ (\Delta T_{it}{}^{Win})^T\ \beta_1 +\ (T_i^{Win})^T\ \beta_2\ + Z_i^T \gamma + \varphi_i + \delta_t +\ \xi_{it}\ + ln(E_{it})$$

Where μ_{it} is the expectation of the observed VL cases at province i at period t, ΔT_{it}^{Win} is the winter temperature anomaly recorded in province i in year t, X_i^T is the 2009-2016 average of winter temperatures recorded in province i, Z_i are baseline explanatory spatial covariates (HIV hospital admission rates, roughness, NDVI), E_{it} are the expected number of VL cases, φ_i and δ_t are the overall spatial and temporal main effects respectively, and ξ_{it} the space-time interaction effect. The spatial random effect, φ_i, depends on ρS parameter defined as in the spatial model. The temporal random effect, $\delta = (\delta_1, \ldots, \delta_8)$ depends on a constant ρT ,that ranges from 0 to 1, and models the temporal correlation between consecutive years. Last, the interaction component, $\xi = (\xi_1, \ldots, \xi_{110*8})$, is a set of space-time identical and independently distributed effects.

Prior specification for the spatial effect:

$$\varphi_i | \varphi_j\ , W \sim N\left(\frac{\rho S \sum_{j=1}^{I} w_{ij} \varphi_j}{\rho S \sum_{j=1}^{I} w_{ij}\ +\ 1\ -\ \rho S}\quad ,\quad \frac{\tau_S^2}{\rho S \sum_{j=1}^{I} w_{ij}\ +\ 1\ -\ \rho S}\right)$$

Prior specification for the temporal effect:

$$\delta_t | \delta_j\ , D \sim N\left(\frac{\rho T \sum_{j=1}^{I} d_{tj} \varphi_j}{\rho T \sum_{j=1}^{T} d_{tj}\ +\ 1\ -\ \rho T}\quad ,\quad \frac{\tau_T^2}{\rho T \sum_{j=1}^{T} d_{tj}\ +\ 1\ -\ \rho T}\right)$$

Prior specification for the spatio-temporal effect:

$$\xi_{it} \sim N(0, \tau_I^2)$$

With:

$$\tau_S^2, \tau_T^2, \tau_I^2 \sim InvGamma(1{,}0.01)$$

$$\rho S, \rho T \sim Uniform\ (0{,}1)$$

References

[1] Lee, D., 2013. CARBayes: An R Package for Bayesian Spatial Modeling with Conditional Autoregressive Priors. J. Stat. Softw. 55, 1–24. https://doi.org/10.18637/JSS.V055.I13

[2] Leroux, B.G., Lei, X., Breslow, N., 2000. Estimation of Disease Rates in Small Areas: A new Mixed Model for Spatial Dependence 179–191. https://doi.org/10.1007/978-1-4612-1284-3_4

[3] Lee, D., Rushworth, A., Napier, G., 2018. Spatio-Temporal Areal Unit Modeling in R with Conditional Autoregressive Priors Using the CARBayesST Package. J. Stat. Softw. 84, 1–39. https://doi.org/10.18637/JSS.V084.I09
